# Heme oxygenase-1 gene promoter polymorphisms are associated with coronary heart disease and restenosis after percutaneous coronary intervention: a meta-analysis

**DOI:** 10.18632/oncotarget.13118

**Published:** 2016-11-04

**Authors:** Ming-Ming Zhang, Ying-Ying Zheng, Ying Gao, Jing-Zhan Zhang, Fen Liu, Yi-Ning Yang, Xiao-Mei Li, Yi-Tong Ma, Xiang Xie

**Affiliations:** ^1^ Department of Cardiology, Luoyang Central Hospital Affiliated to Zhengzhou University, Luoyang 471000, China; ^2^ Heart Center, First Affiliated Hospital of Xinjiang Medical University, Urumqi, Xinjiang 830011, China; ^3^ Department of Cadre ward, First Affiliated Hospital of Xinjiang Medical University, Urumqi, Xinjiang 830011, China

**Keywords:** coronary heart disease, restenosis, heme oxygenase-1 gene, polymorphism, meta-analysis

## Abstract

Numerous published studies have suggested that there is association between heme oxygenase-1 (HO-1) gene polymorphisms and coronary heart disease (CHD) or restenosis (RS) after percutaneous coronary intervention (PCI). This study aimed to clarify this association using a meta-analysis method. We used a systematic search for studies on the association of HO-1gene polymorphisms with CHD or RS in PubMed, Web of Science, the Cochrane Library, Wanfang Data and CNKI (China National Knowledge Infrastructure). We used Stata 12.0 software to perform the meta-analyses. Twenty-three studies, involving 12,130 patients with CHD or RS and 14,181 controls, were included. A statistically significant association between the HO-1(GT)n repeat length polymorphism and CHD was observed under allelic (odds ratio (OR) = 0.929, 95% confidence interval (CI) = 0.881-0.978, *p*= 0.005), recessive (OR = 0.858, 95%CI = 0.780-0.945, *p*= 0.002), and co-dominant (OR = 0.843, 95%CI = 0.754-0.942, *p*= 0.003) models. Moreover, we also found a statistically significant association between the HO-1(GT)n repeat length polymorphism and RS under allelic (OR = 0.718, 95%CI = 0.541-0.953, *p*= 0.022) and co-dominant (OR = 0.522, 95%CI = 0.306-0.889, *p*=0.017) models. We found a significant association of the HO-1T(−413)A single-nucleotide polymorphism (SNP) with CHD under allelic (OR = 0.915, 95%CI = 0.842-0.995, *p*= 0.038), recessive (OR = 0.869, 95%CI = 0.760-0.994, *p*= 0.041), and co-dominant (OR = 0.792, 95%CI = 0.663-0.946, *p*=0.010) models. Our study indicates that both the HO-1(GT)n repeat length polymorphism and the T(−413)A SNP are associated with decreased risk of CHD. The (GT)n repeat length polymorphism was associated with RS following PCI.

## INTRODUCTION

Coronary heart disease (CHD) is a multifactorial disorder resulting from the interaction between environmental and genetic factors [1]. Many genes that associate with CHD have been identified in recent years. In the treatment of CHD, percutaneous coronary intervention (PCI) is the main therapy. However, restenosis (RS) following coronary stenting is a disadvantage of this therapy [2]. Current studies suggest that there are associations between genetic factors and the development of CHD or RS after PCI [3, 4].

Current studies have documented the interaction between HO-1 gene polymorphisms and CHD or RS after PCI. HO-1 is a subtype of heme oxygenase (HO) which plays a key regulatory role in the synthesis and catabolism of bilirubin [5]. HO will be increased significantly when the body responds to oxidative stress. During the degradation of heme to biliverdin, HO plays an important role as a rate-limiting enzyme [6]. Recently, two loci of HO-1 gene have been identified to be associated with CHD or RS in different population [7, 8]. One is the (GT)n dinucleotide repeat length polymorphism, the other is the T(−413)A (rs2071746). Both loci are located in the HO-1 gene promoter region and influence serum HO-1 expression levels [8].

Although many studies on the relationship between these two loci and CHD have been carried out [9–20], the results are not conclusive. Some studies [9–16] have indicated that there is a positive correlation between the HO-1 (GT)n repeat length polymorphism and CHD, while other studies [17–20] have suggested that alterations in HO-1 expression play no obvious role in the pathogenesis of CHD. Several studies [22–23] have indicated that HO-1 genetic polymorphisms are associated with RS after PCI. However, the results of the subsequent studies [17, 24–26] do not support this result.

Based on these observations, two meta-analyses [27–28] related to this topic have been published. Qiao et al. [27] reported a positive correlation between genetic polymorphisms of HO-1 gene and CHD or RS after PCI. However, the meta-analysis from Rong et al. [28] do not support this results. Thus, the association of HO-1 gene polymorphisms with CHD or RS remains unclear. To clarify these associations, we performed an updated meta-analysis.

## RESULTS

### Study characteristics

There were 176 potentially relevant papers acquired from PubMed, Web of Science, the Cochrane Library, Wanfang and CNKI databases. Of these, we excluded 143 documents because of irrelevance to the aim of our study after reading the title and abstract. The remaining 33 documents were full-text reviewed, and 3 studies were excluded due to reported associations with diabetes [29–31]. Four studies were not case-control studies [32–35], 3 studies were excluded because the variable number tandem repeat was different [36–38], 2 studies were excluded because there was no genotype data or it was a review [39–40]. Furthermore, 2 papers were excluded because of their lack of relation to CHD risk but rather to cardiovascular disease prognosis [41–42]. Finally, our meta-analysis included 19 eligible studies [9–26]. Table 1 and Table 2 listed the characteristics of each study. Finally, a total of 13 studies of the (GT)n repeat length polymorphism and 4 studies of the T(−413)A SNP with CHD were included. Six studies of the (GT)n repeat length polymorphism with RS were included. Because 4 papers included 2 studies, there were 23 studies included in final analysis.

**Table 1:** Characteristics of included studies

Reference	Year	Population	Case			Control			Age (years)			Genotyping method	Selection criteria	HWE	VNTR Cut-Off(s) (≥)	NOS (☆)	Study design
**(GT)n polymorphism with CHD**			**Total**	**Male**	**female**	**Total**	**Male**	**female**	**Case**	**Control**	***P***						
Chen et al.	2014	East Asian	386	358	28	361	300	61	70±8	68±8	>0.05	PCR-RFLP	CHD	0.15	27	6	CC
Chen et al.	2012	East Asian	2298	1675	623	2298	1675	623	60.10± 10.3	59.9± 10.2	0.62	CE	CHD	0.11	25	8	CC
Endler et al.	2004	Caucasian	180	130	50	211	103	108	57-72 (64)	48-67 (58)	0.13	PCR-SSP+CE	CHD	0.91	25	6	CC
Funk et al.	2004	Caucasian	399	187	212	398	192	206	59-78 (69)	40-59 (47)	<0.05	PCR-SSP	CHD	0.90	25	6	CC
Gregorek et al.	2013	Caucasian	59	NA	NA	58	NA	NA	62-73 (69)	57-73 (64)	>0.05	PCR-SSP	CHD	0.85	25	6	CC
Han et al.	2014	East Asian	110	71	39	107	56	51	63± 11	52±12	<0.01	PCR-SSP	CHD	0.06	25	6	CC
Kaneda et al.	2002	East Asian	298	250	48	279	173	106	63± 0.5	58±0.7	>0.05	PCR-SSP	CHD	0.32	27	8	CC
Lüblinghoff et al.	2009	Caucasian	2526	1891	635	693	360	333	63± 10	55±12	>0.05	CE	CHD	0.73	25	7	CC
MI in Endler et al.	2004	Caucasian	258	199	59	211	103	108	53-71(60.5)	48-67 (58)	0.22	PCR-SSP+CE	MI	0.91	25	6	CC
Schillinger et al.	2002	Caucasian	70	51	19	62	20	42	62- 78	61-79	0.40	PCR-SSP+CE	CHD	0.15	25	6	CC
Wang et al.	2009	Middle Asian	287	177	110	190	126	64	58.42± 11.1	58.03± 10. 4	0.34	PCR-SSP	MI	0.82	27	7	CC
Y. H. Chen et al.	2008	East Asian	664	611	53	322	264	58	69± 9	67±7	>0.05	CE	CHD	0.49	27	8	CC
Zhang et al.	2010	East Asian	300	228	72	182	106	76	62.96± 12.1	64.23± 12.1	0.13	CE	CHD	0.98	25	7	CC
**(GT)n polymorphism with restenosis**																	
Exner et al	2001	Caucasian	23	NA	NA	73	NA	NA	60-72 (70)	63-72 (69)	0.10	PCR-SSP	CHD	0.02	25	7	CS
Han et al.	2014	East Asian	18	NA	NA	27	NA	NA	63±11	52±12	<0.01	PCR-RFLP	CHD	0.07	25	6	CS
Klaus et al.	2007	Caucasian	401	NA	NA	956	NA	NA	65.5± 10.8	66.2± 10.7	0.51	PCR-SSP	CHD	0.01	25	7	CS
Schillinger et al.	2004	Caucasian	95	NA	NA	183	NA	NA	61-78 (71)	66-78 (73)	0.37	PCR-SSP	CHD	0.58	25	7	CS
Wijpkema et al.	2006	Caucasian	324	NA	NA	2601	NA	NA	NA	NA	NA	PCR-SSP	CHD	0.17	25	6	CS
Y. H. Chen et al.	2003	East Asian	111	NA	NA	212	NA	NA	70±8	68±9	0.07	CE	CHD	0.89	26	7	CS
**T(−413)A polymorphism with CHD**																	
Lüblinghoff et al.	2009	Caucasian	2526	1891	635	693	360	333	63±10	55±12	>0.05	PCR-RFLP	CHD	0.49	NA	7	CC
MI in Ono et al.	2004	East Asian	393	326	67	1972	946	1026	58.4 ±0.6	59.9± 0.3	0.06	PCR-SSP	MI	0.04	NA	8	CC
Ono et al.	2004	East Asian	204	169	35	1972	946	1026	59.7± 0.8	59.9± 0.3	0.07	PCR-SSP	CHD	0.04	NA	8	CC
Zhang et al.	2010	East Asian	200	168	32	120	100	20	61.17± 5.6	62.68± 6.1	0.23	PCR-RFLP	CHD	0.89	NA	7	CC

**Notes:** CC, case-control study; CS, Cohort study; VNTR, variable number tandem repeat; HWE, Hardy-Weinberg equilibrium; CHD, coronary heart disease; MI, myocardial infarction; NOS, Newcastle-Ottawa Scale; PCR, polymerase chain reaction; RFLP, restriction fragment length polymorphism; SSP, sequence-specific primers; CE, capillary electrophoresis.

**Table 2:** Date characteristics of included studies

Reference	Year	Ethnicity	Case						Control					
			N	Genotype (n)			allele		N	Genotype (n)			allele	
				SS	SL	LL	S	L		SS	SL	LL	S	L
**(GT)n polymorphism with CHD**														
Chen et al.	2014	East Asian	386	94	187	105	375	397	361	78	194	89	350	372
Chen et al.	2012	East Asian	2298	436	1268	594	2140	2456	2298	548	1187	563	2283	2313
Endler et al.	2004	Caucasian	180	12	74	94	98	262	211	16	83	112	115	307
Funk et al.	2004	Caucasian	399	39	180	180	258	540	398	46	177	175	269	527
Gregorek et al.	2013	Caucasian	59	7	35	17	49	69	58	10	29	19	49	67
Han et al.	2014	East Asian	110	10	46	54	66	154	107	7	56	44	70	144
Kaneda et al.	2002	East Asian	298	47	165	86	259	337	279	48	145	86	241	317
Lüblinghoff et al.	2009	Caucasian	2526	286	1070	1170	1642	3410	693	66	302	325	434	952
MI in Endler et al.	2004	Caucasian	258	13	106	139	132	384	211	16	83	112	115	307
Schillinger et al.	2002	Caucasian	70	9	38	23	56	84	62	4	32	26	40	84
Wang et al.	2009	Middle Asian	287	57	128	102	242	332	190	55	93	42	203	177
Y. H. Chen et al.	2008	East Asian	664	147	322	195	616	712	322	74	167	81	315	329
Zhang et al.	2010	East Asian	300	39	145	116	223	377	182	27	86	69	140	224
**(GT)n polymorphism with restenosis**														
Exner et al	2001	Caucasian	23	1	8	14	10	36	73	7	45	21	59	87
Han et al.	2014	East Asian	18	1	5	12	7	29	27	0	14	13	14	40
Klaus et al.	2007	Caucasian	401	45	155	201	245	557	956	109	370	477	588	1324
Schillinger et al.	2004	Caucasian	95	3	33	59	39	151	183	20	86	77	126	240
Wijpkema et al.	2006	Caucasian	324	151	151	22	453	195	2601	1256	1124	221	3636	1566
Y. H. Chen et al.	2003	East Asian	111	11	60	40	82	140	212	54	105	53	213	211
**T(−413)A polymorphism with CHD**				AA	AT	TT	A	T		AA	AT	TT	A	T
Lüblinghoff et al.	2009	Caucasian	2526	893	1181	452	2967	2085	693	246	341	106	833	553
MI in Ono et al.	2004	East Asian	393	64	208	121	336	450	1972	420	930	622	1770	2174
Ono et al.	2004	East Asian	204	32	101	71	165	243	1972	420	930	622	1770	2174
Zhang et al.	2010	East Asian	200	40	137	23	217	183	120	28	80	12	136	104

### Meta-analysis

#### HO-1(GT)n repeat length polymorphism and CHD

**Figure 1:**
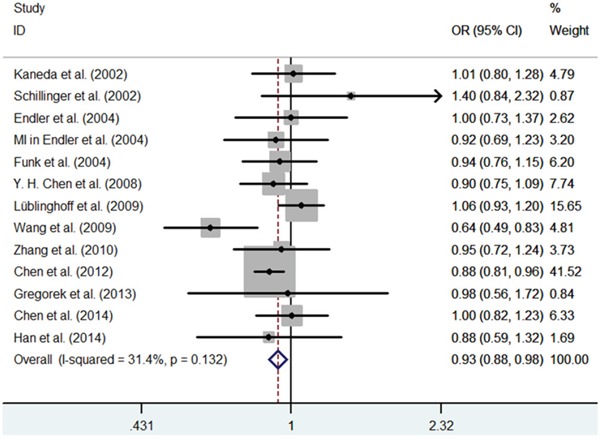
Meta-analysis of the relationship between the (GT)n polymorphism in the HO-1 gene and CHD risk for the allele model (S/L)

**Figure 2:**
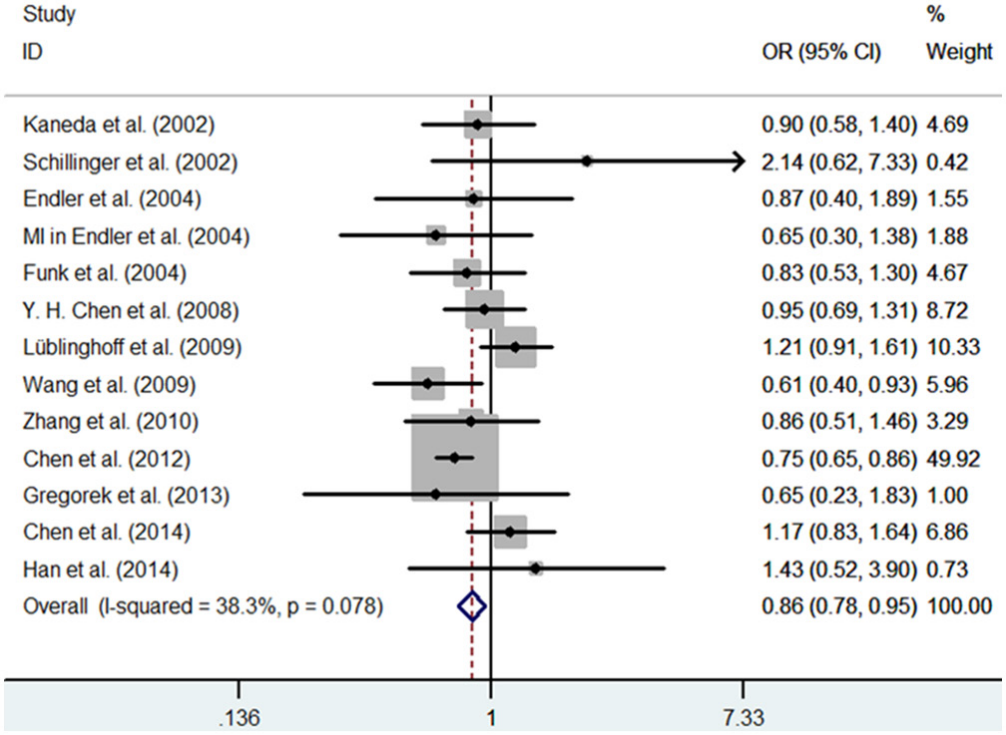
Meta-analysis of the relationship between the (GT)n polymorphism in the HO-1 gene and CHD risk for the recessive model (SS/SL+LL)

**Figure 3:**
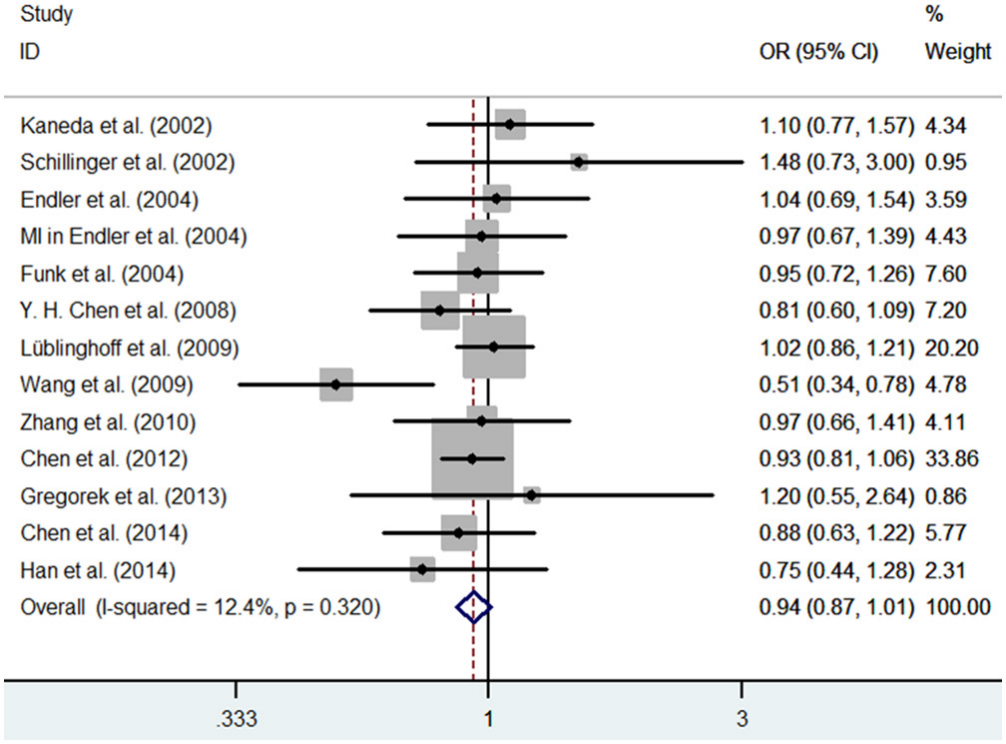
Meta-analysis of the relationship between the (GT)n polymorphism in the HO-1 gene and CHD risk for the dominant model (SS+SL/LL)

**Figure 4:**
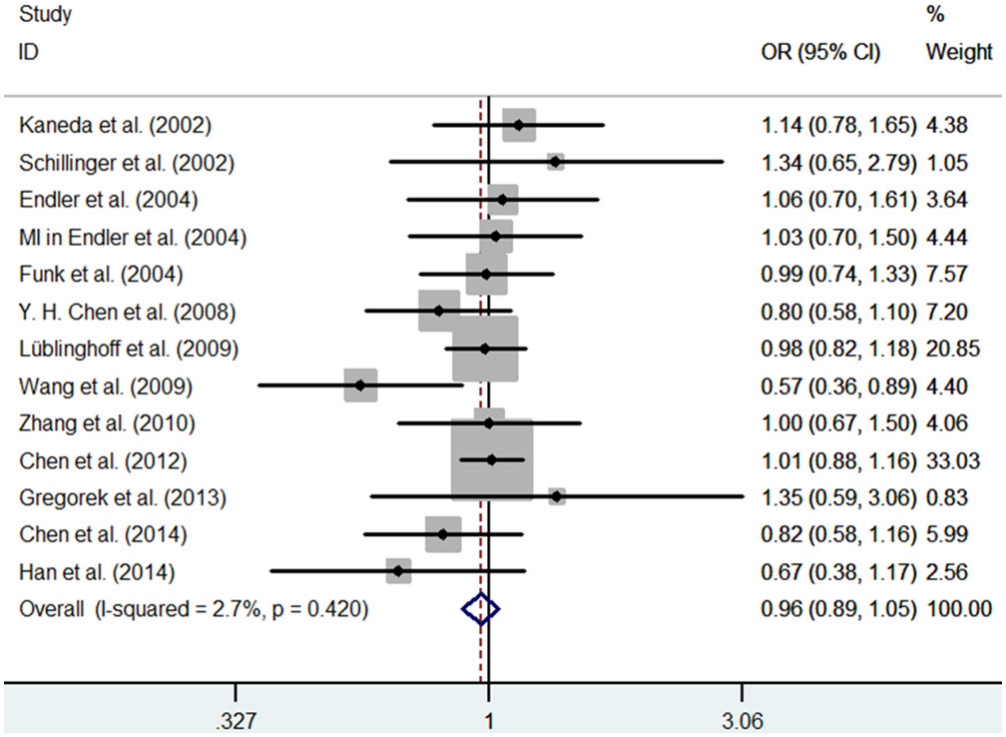
Meta-analysis of the relationship between the (GT)n polymorphism in the HO-1 gene and CHD risk for the co-dominant model (SL/LL)

**Figure 5:**
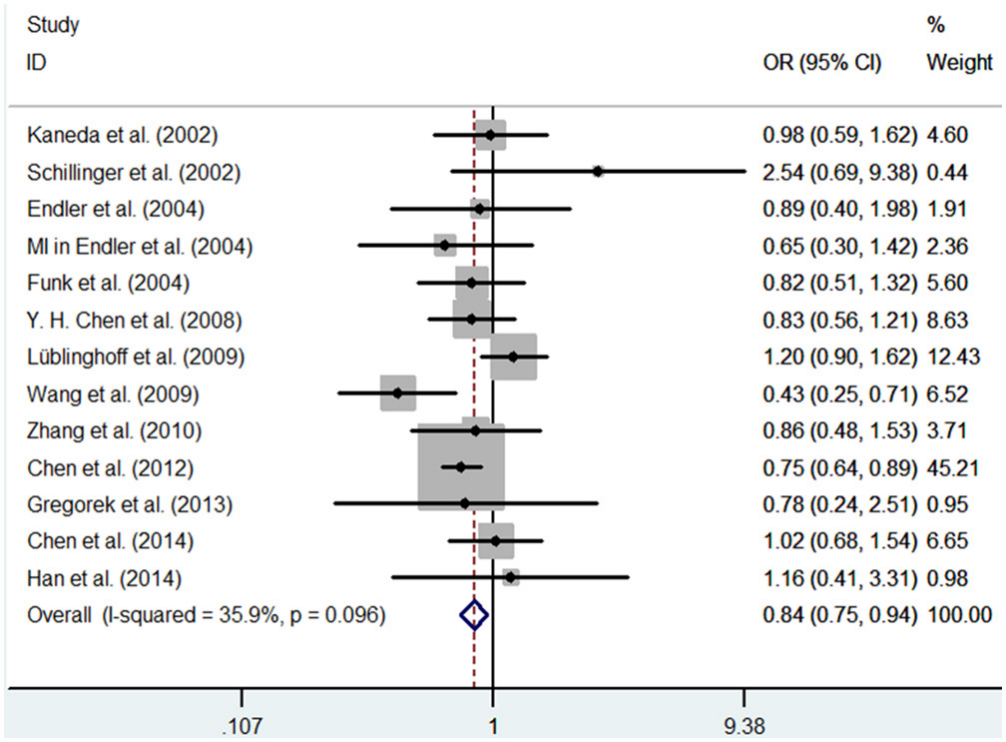
Meta-analysis of the relationship between the (GT)n polymorphism in the HO-1 gene and CHD risk for the co-dominant model (SS/LL)

First, we investigated the relation between HO-1(GT)n repeat length polymorphism and CHD. No significant heterogeneity was identified by H-test and I^2^ test in any of the genetic models (Table 3), therefore, the fixed-effect model was used. Significant statistical association was found between HO-1(GT)n repeat length polymorphism and CHD risk under an allelic contrast (S vs. L, OR = 0.929, 95%CI = 0.881-0.978, P = 0.005), the recessive genetic model (SS vs. SL+LL, OR = 0.858, 95% CI = 0.780-0.945, P = 0.002), and the co-dominant genetic model (SS vs. LL, OR = 0.843, 95% CI = 0.754-0.942, P = 0.003). Comparing to SL+LL and LL genotypes carriers, the CHD risk was significantly decreased among the SS genotype patients (Figure 1–5). However, we did not find significant association in the dominant genetic model (Table 4).

**Table 3:** heterogeneity test analysis -(GT)n repeat length polymorphism with CHD

Study	Heterogeneity test		
	*H*	*I^2^*	*P*
Allele model(S/L)	1.21	31.40%	0.132
Recessive model(SS/SL+LL)	1.27	38.30%	0.078
Dominant model(SS+SL/LL)	1.07	12.40%	0.320
Co-dominant model(SL/LL)	1.01	2.70%	0.420
Co-dominant model(SS/LL)	1.25	35.90%	0.096

**Table 4:** Results From a Meta-Analysis of the Association Between coronary heart disease or restenosis after PCI and the Heme oxygenase-1 gene promoter polymorphism

Polymorphism and Subgroup	No. of Studies	No. of Cases	No. of Controls	Genotype									
				S/L		SS/SL+LL		SS+SL/LL		SL/LL		SS/LL	
				OR and 95%CI	*P* Value	OR and 95%CI	*P* Value	OR and 95%CI	*P* Value	OR and 95%CI	*P* Value	OR and 95%CI	*P* Value
**(GT)n polymorphism with CHD**													
**Total**	13	7835	5372	0.929(0.881, 0.978)	0.005	0.858(0.780, 0.945)	0.002	0.937 (0.867, 1.012)	0.100	0.963(0.888, 1.045)	0.369	0.843 (0.754, 0.942)	0.003
**Caucasian**	6	3492	1633	1.019 (0.927, 1.119)	0.701	1.033(0.840, 1.271)	0.759	1.020 (0.901, 1.154)	0.756	1.015(0.892, 1.156)	0.818	1.042(0.838, 1.296)	0.709
**Asian**	7	4343	3739	0.891 (0.837, 0.949)	0.000	0.815(0.731, 0.909)	0.000	0.887 (0.803, 0.980)	0.018	0.931(0.838, 1.034)	0.180	0.781(0.686, 0.890)	0.000
**Good quality**	6	6373	3964	0.915 (0.863, 0.971)	0.003	0.830 (0.746, 0.924)	0.001	0.929 (0.849, 1.017)	0.110	0.964(0.877, 1.060)	0.551	0.822(0.726, 0.930)	0.002
**Poor quality**	7	1462	1408	0.959 (0.856, 1.074)	0.468	0.995 (0.795, 1.246)	0.976	0.960 (0.824, 1.117)	0.595	0.961(0.819, 1.127)	0.623	0.935(0.728, 1.201)	0.599
**(GT)n polymorphism with restenosis**													
**Total**	6	972	4052	0.718 (0.541, 0.953)	0.022	0.674 (0.425, 1.069)	0.093	0.662(0.434, 1.010)	0.056	0.877(0.740, 1.039)	0.130	0.522(0.306, 0.889)	0.017
**Caucasian**	4	843	3813	0.766 (0.557, 1.053)	0.100	0.870 (0.637, 1.190)	0.384	0.694 (0.400, 1.204)	0.194	0.742 (0.439, 1.254)	0.265	0.72 (0.384, 1.380)	0.330
**Asian**	2	129	239	0.590 (0.430, 0.809)	0.001	0.755(0.065, 0.737)	0.022	0.572 (0.361, 0.907)	0.018	0.689 (0.426, 1.115)	0.130	0.548(0.461, 0.660)	0.003
**Meeting HWE**	4	548	3023	0.679(0.446, 0.934)	0.041	0.553(0.230, 1.327)	0.184	0.664(0.381, 1.156)	0.148	0.740(0.435, 1.258)	0.266	0.414(0.195, 0.879)	0.022
**Deviating from HWE**	2	424	1029	0.693(0.296, 1.620)	0.397	0.959(0.667, 1.380)	0.822	0.554(0.151, 2.034)	0.373	0.566(0.158, 2.209)	0.382	0.684(0.192, 2.434)	0.557
**T(−413)A polymorphism with CHD**				A/T		AA/AT+TT		AA+AT/TT		AT/TT		AA/TT	
**Total**				OR and 95%CI	*P* Value	OR and 95%CI	*P* Value	OR and 95%CI	*P* Value	OR and 95%CI	*P* Value	OR and 95%CI	*P* Value
	4	3323	4757	0.915(0.842, 0.995)	0.038	0.869(0.760, 0.994)	0.041	0.907(0.788, 1.045)	0.177	0.958(0.826, 1.110)	0.567	0.792(0.663, 0.946)	0.010

The second subgroup analysis was conducted according to ethnicity. The fixed-effects model was utilized to perform meta-analysis in all of the genetic models. We found patients with SS genotype have decreased CHD risk compared to SL+LL and LL genotype carriers in the Asian subgroup (S vs. L, OR = 0.891, 95% CI = 0.837-0.949, P = 0.000; SS vs. SL+LL, OR = 0.815, 95% CI = 0.731-0.909, P = 0.000; SS+SL vs. LL, OR = 0.887, 95% CI = 0.803-0.980, P = 0.018; SS vs. LL, OR = 0.781, 95% CI = 0.686-0.890, P = 0.000). However, this association was not observed in Caucasian populations (Table 4).

In addition, we conducted subgroup analysis according to quality assessment. The fixed-effects model was used in all of the genetic models. Significantly decreased risk of CHD was found among individuals with the SS genotype compared to patients with L allele (SL + LL and LL genotypes) in the good-quality subgroup (S vs. L, OR = 0.951, 95% CI = 0.863-0.971, P = 0.003; SS vs. SL+LL, OR = 0.830, 95% CI = 0.746-0.924, P = 0.001; SS vs. LL, OR = 0.822, 95% CI = 0.726-0.930, P = 0.002). However, this association was not found in the poor-quality reports (Table 4).

#### HO-1(GT)n repeat length polymorphism and RS

**Figure 6:**
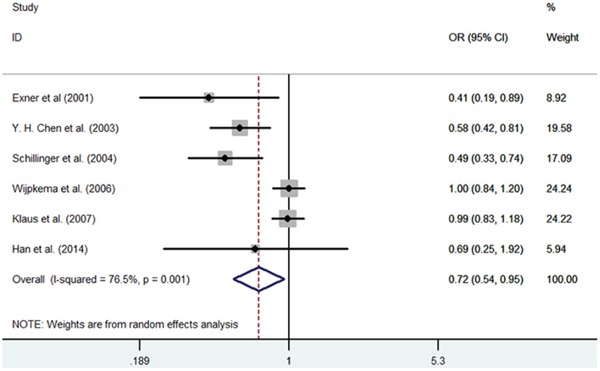
Meta-analysis of the relationship between the (GT)n polymorphism in the HO-1 gene and RS after PCI for the allele model (S/L)

**Figure 7:**
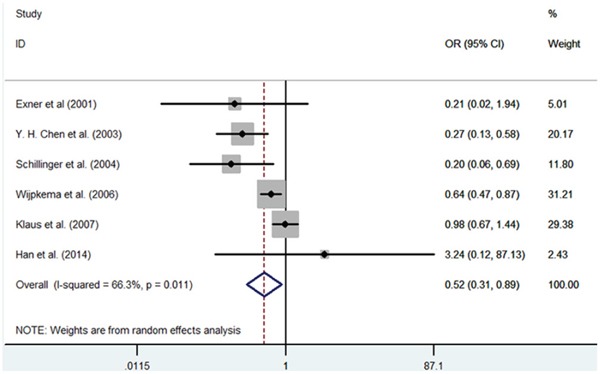
Meta-analysis of the relationship between the (GT)n polymorphism in the HO-1 gene and RS after PCI for the allele model (SS/LL)

In 6 independent studies, drug-eluting stents were utilized. These studies examined the main baseline characteristics and identified no significant difference. First, significant heterogeneity was found in the contrast models, and therefore, the random-effects model was used in this meta-analysis. In the overall population, we found that patients with S allele had a decreased RS risk after PCI compared with the L allele carriers (S vs. L, OR = 0.718, 95% CI = 0.541-0.953, P = 0.022; SS vs. LL, OR = 0.522, 95% CI = 0.306-0.889, P = 0.017) (Figure 6–7). However, we did not found significantly decreased risks of RS in other genetic models (SS vs. SL+LL, OR = 0.674, 95% CI = 0.425-1.069, P=0.093; SS+SL vs. LL, OR = 0.662, 95% CI = 0.434-1.010, P = 0.056; SL vs. LL, OR = 0.877, 95% CI = 0.740-1.039, P = 0.130). Second, subgroup analysis was conducted according to ethnicity. The RS risk was significantly decreased among patients with the SS genotype compared with other genotypes in the Asian subgroup (S vs. L, OR= 0.590, 95% CI = 0.430-0.809, P = 0.001; SS vs. SL+LL, OR = 0.755, 95% CI = 0.065-0.737, P = 0.022; SS+SL vs. LL, OR = 0.572, 95% CI = 0.361-0.907, P = 0.018; SS vs. LL, OR = 0.548, 95% CI = 0.461-0.660, P = 0.003). When we excluded the studies which were inconsistent with the HWE, the protective effects of the S allele for RS after PCI persisted (S vs. L, OR= 0.679, 95% CI = 0.446-0.934, P = 0.041; SS vs. LL, OR = 0.414, 95% CI = 0.195-0.879, P = 0.022) (Table 4).

#### HO-1 T(−413)A SNP and CHD risk

There were 4 studies that investigated the relationship between the HO-1T(−413)A SNP and CHD. No significant heterogeneity was found in the contrast models, and so the fixed-effect model was used in this of meta-analysis. Meta-analysis suggested that there was a significant association between the HO-1 T(−413)A polymorphism and CHD under the allele contrast (A vs. T, OR = 0.915, 95% CI = 0.842-0.995, P = 0.038), the recessive genetic model (AA vs. AT+TT, OR = 0.869, 95% CI = 0.760-0.994, P = 0.041), and the co-dominant genetic model (AA vs. TT, OR = 0.792, 95% CI = 0.663-0.946, P = 0.010) (Table 4).

### Sensitivity analysis

**Figure 8:**
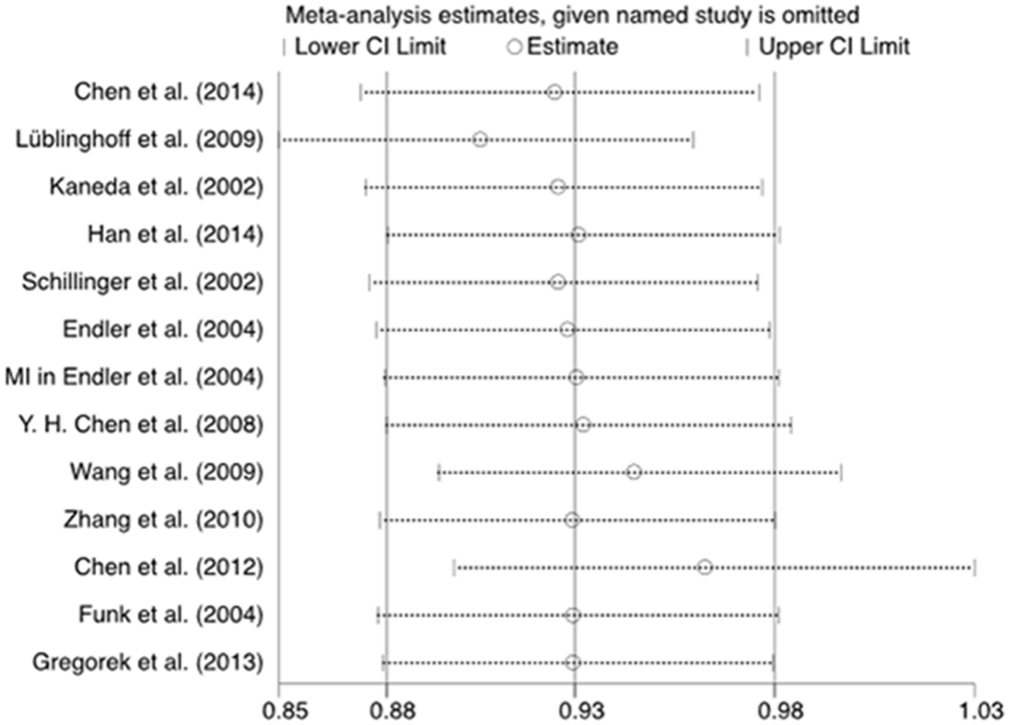
Sensitivity analysis of the relationship between the (GT)n polymorphism in the HO-1 gene and CHD risk for the allele model

We performed the sensitivity analysis to examine the influence of each study on the pooled ORs by deleting each study one at a time in each genetic model. The pooled ORs showed no significant change, suggesting the results are stable (Figure 8).

### Publication bias

**Figure 9:**
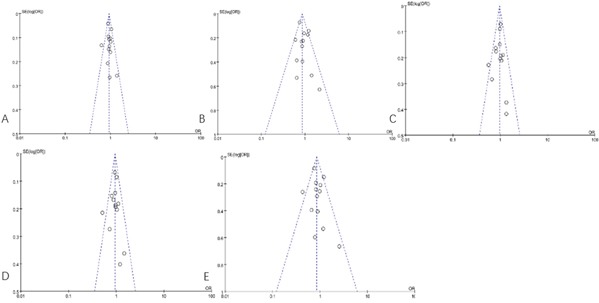
Funnel plot of the association between the (GT)n polymorphism in the HO-1 gene and CHD risk **A.** the allele model (S/L); **B.** the recessive model (SS/SL+LL); **C.** the dominant model (SS+SL/LL); **D.** the co-dominant model (SL/LL); **E.** the co-dominant model (SS/LL).

In the present study, we utilized Egger's test and funnel plots to evaluate the publication bias of all contrast models. By Egger's test and funnel plots, we did not found publication biases for both the (GT)n repeat length polymorphism and T(−413)A SNP (Table 5, Figure 9).

**Table 5:** Egg's test results

Association	Genetic model	*P* value
(GT)n polymorphism and CHD	S versus L	0.598
	SS versus SL+LL	0.301
	SS+SL versus LL	0.823
	SL versus LL	0.975
	SS versus LL	0.519
(GT)n polymorphism and RS after PCI	S versus L	0.068
	SS versus SL+LL	0.366
	SS+SL versus LL	0.127
	SL versus LL	0.133
	SS versus LL	0.142
T(−413)A polymorphism and CHD	A versus T	0.395
	AA versus AT+TT	0.263
	AA+AT versus TT	0.820
	AT versus TT	0.909
	AA versus TT	0.370

## DISCUSSION

The present study indicates that HO-1 gene polymorphisms are associated with CHD independently. There are three isoforms of heme oxygenase in human, including HO-1, HO-2 and HO-3. HO-1 is up-regulated by oxidative stress and its own substrate heme [43] and may be modulated by fragile histidine triad gene (*FHIT*) [44]. Animal experiments and clinical trials have confirmed that the HO-1 enzyme is expressed in various tissues and cells, including asatherosclerotic lesions and vascular smooth muscle cells [43]. Therefore, HO-1 is considered to provide protective functions against asatherosclerotic lesions formation [43] and cellular proliferation [45].

Recently, many studies have suggested that HO-1 gene polymorphisms were associated with CHD [9–20]. The SS genotype of the HO-1(GT)n locus may promote HO-1 gene expression and result in increased protein production, thereby raising bilirubin levels and so reducing the risk of CHD [9–16] and restenosis following coronary stenting [22, 23]. However, some studies have come to other conclusions. Theoretically, meta-analysis can clarify the conclusions. Unfortunately, the two previously published meta-analyses came to different conclusions. Recently several new publications focused on this topic have been published. We conducted an update meta-analysis to clarify the association of HO-1 polymorphisms with CHD and RS.

In the present study, a significant association of the (GT)n SS genotype or S allele with decreased the risk of CHD and RS after PCI was observed. In the subgroup analysis, the Asian population showed a positive association in the all genetic models, while the study conducted with the Caucasian population showed no significant association in any of the genetic models. This can be explained by the high prevalence of S allele in Asian subjects. In addition, because of difference in life styles, ethnicity, region, and other factors, there are large differences in gene distribution. Nevertheless, more studies have been included in the present meta-analysis, and all the included studies were of high quality according to the methodological quality assessment. No significant heterogeneity was identified, and supplementary analysis, including subgroup and sensitivity analysis, were performed to strengthen our conclusions.

The present study also shows an association between genetic factors and the risk of stenting RS. We found that the HO-1T(−413)A SNP was associated with decreased risk for CHD. However, this significant association should be interpreted cautiously. First, CHD or RS after PCI are complex diseases with multifactorial etiology, including gene and environmental factors. Only one SNP is not sufficient to provide the appropriate explanation of genetic risk for CHD or RS after PCI. Gene-gene or gene-environment interaction factors may influence the risk of a subject for CHD or RS. Second, some potentially confounding factors should be discussed. Primary sources of heterogeneity include the following: the condition of the population included in this study, the main characteristics of the stents following PCI and the treatment compliance of the patients. In addition, the number of included studies for the HO-1T(−413)A SNP is small, and so we did not perform further subgroup analysis in the present study. Even so, the conclusion still give us some information on the pathogenesis of CHD and RS risk factors. Indeed, HO-1 is involved in the occurrence of restenosis by inhibiting vascular smooth muscle cells, attenuating vascular remodeling, and other mechanisms [48, 49]. Although in our meta-analysis, we found that S allele carriers have decreased risk for RS after PCI compare with L allele carriers and that the HO-1 T(−413)A SNP was associated with decreased risk of CHD, the importance of HO-1 in human RS following coronary stenting has not been fully defined.

Several limitations of our study need to be considered. First, the number of included studies for HO-1T(−413)A SNP is small, and so we did not perform further subgroup analyses in the present study. Second, limiting the included studies to those published in English and Chinese might have missed some eligible studies in other languages. In addition, it is possible that the results included in the present meta-analysis are affected by miscounting the genotypes or misclassification of CHD and RS.

Hence, our results suggested that carrying the S allele of the (GT)n locus or the A allele of the T(−413)A locus in the HO-1 gene promoter decreased the risk of CHD. We also found that carrying shorter (GT)n repeats (S or SS genotype) but not the T(−413)A SNP was associated with decreased risk of RS after PCI. These effects appeared more significant in Asian populations.

## MATERIALS AND METHODS

### Identification of eligible studies

We carried out a systematic search in PubMed, Web of Science, the Cochrane Library, Wanfang Data and CNKI (China National Knowledge Infrastructure), with the last search updated on February 1, 2016. The following terms were used: “heme oxygenase 1” or “HO-1” or “HMOX-1” and “polymorphism” and “coronary artery disease” or “cardiac heart disease” or “myocardial infarction” or “MI” or “angina pectoris” or “arteriosclerosis” or “coronary disease” or “acute coronary syndrome” or “coronary stenosis” or “restenosis” or “stent-restenosis”. We included literature on relevant studies carried out in human subjects published in English and Chinese. CHD was defined as confirmed myocardial infarction, typical angina pectoris (by the World Health Organization criteria), or a history of PCI or as diagnosed by angiography. The controls were defined as in-patients, outpatients, or members of the general population who were without typical angina pectoris or electrocardiographic abnormality and without coronary artery stenosis of more than 20% upon coronary angiography [28].

### Inclusion criteria

The studies in our meta-analysis met the following inclusion criteria: (1) case-control or cohort studies; (2) investigation of the association between the HO-1 gene polymorphisms and coronary artery disease or coronary restenosis; (3) inclusion of all patients, which were using drug-eluting stents(DES) and had 6 months follow-up angiography, after stenting (Restenosis, was defined as angiographic restenosis, diameter stenosis of >50%, and clinical restenosis, target vessel revascularization during the follow-up.);(4) studies focusing on humans; and (5) unabridged genotype data could be acquired to calculate the odds ratios (ORs) and 95% confidence intervals (CIs).

### Exclusion criteria

We excluded papers according to the following criteria: (1) studies with no genotype data; (2) commentaries, reviews and editorials; (3) family-based studies of pedigrees; and (4) repeated studies using the same population data or duplicated data.

### Data extraction

Data collection from the eligible studies were conducted independently by two investigators (Zhang and Zheng). The following contents were collected: name of the first author, year of publication, ethnicity or geographic location of the study subjects, the characteristics of cases and controls, genotyping methods, number of cases and controls, the criteria for cases and controls, genotype frequency in cases and controls for HO-1 genotypes, Hardy–Weinberg equilibrium, and type of stents. Two investigators checked the extracted data and reached a consensus on all the data. If a disagreement existed, a third investigator (Xie) would adjudicate the disagreement.

### Quality assessment

To determine the methodological quality of each study, we used the Newcastle-Ottawa scale (NOS), which uses a “star” rating system to judge the quality of all included studies. The NOS ranges between zero (worst) and nine stars (best). Studies with a score equal to or higher than seven were considered to be of good quality. A score equal to or higher than four and less than seven was regarded as being poor quality. Two investigators (Zheng and Zhang) independently assessed the quality of included studies, and the results were reviewed by a third investigator (Xie). Disagreement was resolved by discussion.

### Statistics analysis

Stata 12.0 software (StataCorp, College Station, TX, USA) was used for statistical analysis in our meta-analysis. The Hardy–Weinberg equilibrium (HWE) was calculated for each study using the Chi-square test in control groups, and P < 0.05 was considered a significant deviation from the HWE. Odds ratios and 95% confidence intervals were calculated to assess the strength of the associations of the polymorphism and susceptibility to CHD or RS risk. The associations between the genetic variant and CHD or RS risk of pooled ORs were performed for allelic comparison [(GT) n: S vs. L; T(−413)A: A vs. T], a recessive genetic model [(GT) n: SS vs. SL+LL; T(−413)A: AA vs. AT+TT], a dominant model [(GT) n: SS+SL vs. LL; T(−413)A: AA+AT vs. TT], and a co-dominant model [(GT) n: SL vs. LL, SS vs. LL; T(−413)A: AT vs. TT, AA vs. TT]. The statistically significant level was determined by Z-test, and significance was set at *p*<0.05. Heterogeneity was assessed using the H test (significance level of *P*< 0.1) and the I^2^ test (greater than 50% as evidence of significant inconsistency). Pooled effect sizes were determined using a fixed-effects model (the Mantel–Haenszel method) when heterogeneity was negligible (I^2^<50%) or a random-effects model (the DerSimonian and Kacker method) when significant heterogeneity was present (I^2^≥50%). We also performed a sensitivity analysis to evaluate the effect of each study on the combined ORs by omitting each study in turn. Finally, we utilized Egger's tests to assess the potential publication bias.
